# Evaluation of Aromatic Plants and Compounds Used to Fight Multidrug Resistant Infections

**DOI:** 10.1155/2013/525613

**Published:** 2013-10-08

**Authors:** Ramar Perumal Samy, Jayapal Manikandan, Mohammed Al Qahtani

**Affiliations:** ^1^Infectious Diseases Programme, MD4, 5 Science Drive 2, Department of Microbiology, Yong Loo Lin School of Medicine, National University Health System (NUHS), National University of Singapore, Singapore 117597; ^2^Center of Excellence in Genomic Medicine Research, King Abdulaziz University, P.O. Box 80216, Jeddah 21589, Saudi Arabia; ^3^School of Anatomy, Physiology and Human Biology, The University of Western Australia, 35 Stirling Highway, Crawley, WA 6009, Australia

## Abstract

Traditional medicine plays a vital role for primary health care in India, where it is widely practiced to treat various ailments. Among those obtained from the healers, 78 medicinal plants were scientifically evaluated for antibacterial activity. Methanol extract of plants (100 **μ**g of residue) was tested against the multidrug resistant (MDR) Gram-negative and Gram-positive bacteria. Forty-seven plants showed strong activity against *Burkholderia pseudomallei* (strain TES and KHW) and *Staphylococcus aureus*, of which *Tragia involucrata* L., *Citrus acida* Roxb. Hook.f., and *Aegle marmelos* (L.) Correa ex Roxb. showed powerful inhibition of bacteria. Eighteen plants displayed only a moderate effect, while six plants failed to provide any evidence of inhibition against the tested bacteria. Purified compounds showed higher antimicrobial activity than crude extracts. The compounds showed less toxic effect to the human skin fibroblasts (HEPK) cells than their corresponding aromatic fractions. Phytochemical screening indicates that the presence of various secondary metabolites may be responsible for this activity. Most of the plant extracts contained high levels of phenolic or polyphenolic compounds and exhibited activity against MDR pathogens. In conclusion, plants are promising agents that deserve further exploration. Lead molecules available from such extracts may serve as potential antimicrobial agents for future drug development to combat diseases caused by the MDR bacterial strains as reported in this study.

## 1. Introduction 

Treatment of infections is compromised worldwide by the emergence of bacteria that are resistant to multiple antibiotics [1]. New and emerging drug resistance bacteria strains, particularly methicillin-resistant *Staphylococcus aureus* (MRSA), vancomycin-resistant enterococci (VRE), *Mycobacterium tuberculosis* (MTB), and multidrug resistance (MDR) Gram-negative bacteria, are increasing worldwide and add to the gravity of the situation [2]. *S. aureus* cause a variety of syndromes such as food poisoning, toxic shock syndrome, skin lesions [3], hyperproliferative skin disease [4], and atopic dermatitis [5, 6]. Community-acquired pneumonia caused by *Streptococcus pneumoniae, Klebsiella pneumonia, *and* S. aureus* accounts for significant mortality in Southeast Asia [7]. Melioidosis has been recognized as an important human infection caused by *Burkholderia pseudomallei* in Singapore, Malaysia, Thailand, and Northern Australia [8, 9]. Cases have also been reported from some other tropical and subtropical regions like Africa and America, and a number of cases in man has recently been reported to increase in China, Taiwan, and South India [10, 11]. Infection with antibiotic resistant bacteria negatively impacts on public health, due to an increased incidence of treatment failure and severity of diseases. Development of resistant bacteria due to the chromosomal mutations is more commonly associated with the horizontal transfer of resistance determinants borne on mobile genetic elements [12]. *B. pseudomallei* is intrinsically resistant to many antibiotics [13, 14]. Considering the higher cost for producing synthetic drugs and the various side effects associated with their use, the need to search for alternative agents from medicinal plants and essential oils used in folklore medicine is further justified to overcome these issues. In India, there are about 550 tribal communities covered under 227 ethnic groups residing in about 5000 villages throughout different forest and vegetation regions [15]. India is one of the world's 12 megabiodiversity countries [16, 17]. Plant derived medicines have played a major role in human societies throughout the history and prehistory of mankind [18]. The traditional healers (traditional physicians) or medicinemen have a long history of their own diagnostic and treatment system, which they have acquired from their ancestors [19]. Approximately 80% of the world population still relies on traditional medicine for the treatment of common diseases [20–22]. Medicinal plants thus offer significant potential for the development of novel antibacterial therapies and adjunct treatments [23]. Plant derived drugs serve as a prototype to develop more effective and less toxic medicines. In previous studies, few attempts were made to confirm the antimicrobial activity of some indigenous medicinal plants [24, 25]. Not only extracts of various medicinal plants but also essential oils and their constituents have been investigated for their antimicrobial properties against bacteria and fungi [26–28].

The principal compounds from the leaves showed a better antibacterial activity against *P. aeruginosa* and *B. subtilis* bacteria and a significant antifungal activity on *C. albicans *[29]. The essential oil from *R. officinalis* (alpha pinene/verbenone/bornyl acetate) was found to be more sensitive to the Gram-positive bacteria (MIC 2.5–4 mg/mL) than to the Gram-negative bacteria [30]. Several essential oils kill bacteria by damaging the cell membrane structure and inhibiting their membrane function [31]. Because of the antimicrobial potency of plant extracts and oils, they become a rich source of raw materials for many biotechnological and pharmaceutical industries for the development of therapeutic drugs. The increasing trend in the use of aromatic plants and essential oils in food, cosmetic, and pharmaceutical industries suggests that a systematic study of traditional medicinal plants is very important in order to find active compounds from such sources [32–34]. The purpose of this study is to survey and investigate popular medicinal aromatic plants and their essential oils with a view to fight against multidrug resistant human pathogens. In the present study, 71 plant species were selected on the basis of the available medicinal information and screened for their *in vitro* antimicrobial efficacy against bacteria.

## 2. Materials and Methods

### 2.1. Ethnomedicinal Survey and Collection of Plants

Ethnomedicinal surveys were conducted during March 1998 and July 2001 from various tribal localities (Kolli hills, Kalrayan hills, Pachamalai, Javadi hills, Mundanthurai) of Eastern and Western Ghats, Tamil Nadu, India. For ethnobotanical studies, questioners were used to collect the general information on the tribes, and the key information on medicinal details was collected through interviews. The medicinal plants were identified by a taxonomist using the standard Flora of Tamil Nadu Carnatic [35], and the voucher specimens were deposited in the department's herbarium at the Entomology Research Institute, Loyola College, Chennai, India. 

### 2.2. Preparation of Plant Extracts

Using a Soxhlet apparatus, the shade-dried and powdered plant materials (200 g of each) were extracted with 1000 mL of methanol (CH_3_OH) for 10 h. The collected methanol extracts were filtered (Whatman no. 1 filter paper) and evaporated with a rotary evaporator and freeze dryer (lyophilized) to obtain the crude extracts (Buchi, Labortechnik AG, Switzerland). The dried crude extracts were stored at 4°C for antimicrobial assays [34]. 

### 2.3. Culture of Microorganisms

The following Gram-negative: *Burkholderia pseudomallei* (TES21), *Burkholderia pseudomallei* (KHW22), *Klebsiella pneumoniae* (ATCC15380), *Klebsiella pneumoniae*, *Pseudomonas aeruginosa* (ATCC27853), *Vibrio damsela*, and *Salmonella typhi* (ATCC51812) and Gram-positive: *Staphylococcus aureus* (ATCC 29213), *Streptococcus pyogenes*, and *Streptococcus pneumoniae* (ATCC49619) microorganisms were used for cultures. *B. pseudomallei* bacterial strains such as KHW and TES were isolated from the patient samples obtained from the Department of Microbiology, NUS, Singapore. The strains were subcultured on 20 mL Tryptic Soy (TS) and Mueller Hinton (MH) agar plates (pH 7.4) and incubated overnight at 37°C before use.

### 2.4. Antimicrobial Activity

The standard bacterial cultures were stored at −70°C, subcultured on 20 mL MH and TS agar plates (pH 7.4), and incubated overnight at 37°C prior to use. The antimicrobial property was tested using the disc-diffusion method [36]. Five young colonies of each strain of bacteria taken from their respective cultures grown overnight on TS agar plates (Oxoid limited, Wode Road, Basingstoke, Hants, England, UK) were suspended in 5 mL of sterile saline (0.9%), and the density of the suspension was adjusted to approximately 3 × 10^8^ colony forming unit (CFU). The swab was used to inoculate the dried surface of TS agar plate by streaking four times over the surface of the agar and rotating the plate approximately 90°C to ensure an even distribution of the inoculums. The medium was allowed to dry for about 3 min before adding a 6 millimeter in diameter (mm) sterile paper disc (Becton Dickinson, USA) on the surface. Each disc was tapped gently down onto the agar to provide a uniform contact. Lyophilized residue (100 *μ*g/mL) of each plant extracts and purified fractions was weighed and dissolved in 1 mL of water, and 20 *μ*L of the extracts and oils (containing 100 *μ*g of residue) were applied on each disc (3 replicates), while the sterile blank disc served as a normal control. The antimicrobial effect of the extracts on the clinical isolates was determined in comparison with the reference antibiotics (chloramphenicol 30 *μ*g/disc and ceftazidime 30 *μ*g/disc), which were used as positive controls. The plates were incubated at 37°C for 24 h, and the inhibition zones were measured and calculated. 

### 2.5. Minimum Inhibitory Concentrations (MICs) Assay

MICs were evaluated based on the *in vitro* screening of 16 purified fractions that were found to have potent antimicrobial activity. Broth dilution method was used for the MIC assay with some slight modifications as recommend by the NCCLS [37]. Two-fold serial dilutions of all the fractionated compounds were made with MH and TS broth in microtiter plate wells to adjust the final concentration from 7.8 to 250 *μ*g/mL, while wells containing the broth alone without any sample served as a control. Three replicates (*n* = 3) were used for each dilution and culture containing approximately 1 × 10^5^ CFU/mL. The plates were incubated at 37°C for 24 h, and the absorbance was measured at 560 nm.

### 2.6. Cytotoxicity Assay

The cytotoxic effects of various extracts were tested by MTT assay [38] using human skin fibroblast HEPK cells. The toxic effect of plant extracts and essential oils was assayed on human skin fibroblast (HEPK) cell proliferation in 96-well microtitre plates. Confluent cells (5 × 10^6^ cells per well) were incubated with extracts and oils for 24 h, and the percentage inhibitory concentration (IC_50_) was determined. 

### 2.7. Phytochemical Analysis of Plant Extracts

The most active extracts were used for purification of antimicrobial compounds [39]. 100 g each of the plant powder was percolated with 500 mL of 4% aqueous HCl (adjusted to pH 2) and heated at 50°C for 3 h. The extract was washed with 2 × 250 mL of diethyl ether, and the organic phase evaporated to dryness using vacuum rotary evaporator. The dark brown gummy residues obtained by acid hydrolysis were chromatographed on silica gel column 60 × 3.2 cm (60–120 mesh, pH 7, Merck) by gradually eluting with n-hexane/ethyl acetate (8 : 2; 6 : 4; 3 : 7 and 1 : 1) and chloroform/methanol (3 : 2). The aliquots of each fraction were subjected to thin layer chromatography (TLC) on silica gel coated TLC plate (1 mm Merck) using the solvent system consisting of 20% (v/v) n-hexane/ethyl acetate. The chromatograms were detected using 50% H_2_SO_4_ solution as a spray reagent [34]. The individual fractions were collected and concentrated by vacuum rotary evaporator at 40°C. All the purified compounds recovered from the silica gel column were monitored by reading the absorbance at 190–350 nm (UV spectrophotometer, Hitachi, Japan). The active fractions were further purified, and the final yields of the compounds were recorded. The lyophilized pooled concentrated compounds were then assayed (100 *μ*g/mL) against bacteria. The phytochemical screening was done on the pure compounds using the chemical method previously reported for the detection of secondary metabolites [40]. The different chemical constituents tested include alkaloids, flavonoids, glycosides, polyphenols, saponin, sterols, triterpenes, tannins, reducing sugars, gallic acid, catechol, and aglycones.

### 2.8. Statistical Analysis

The bacterial growth inhibitory activity (inhibition zones millimeter in diameter) was compared for significant differences within the bacterial strains. One way analysis of variance was performed (mean ± SD, *n* = 3 replicates) using GraphPad Prism 4, USA. *P < 0.01 was considered statistically significant (inhibition zones of extracts/fractions versus antibiotic drugs).

## 3. Results

### 3.1. Study Area for the Collection of Aromatic Medicinal Plants

**Figure 1:**
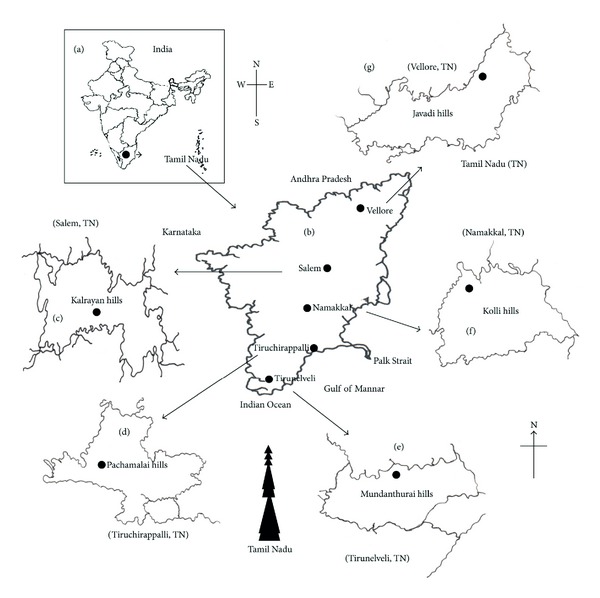
(a) The site for collection of medicinal plants in Western and Eastern Ghats of Tamil Nadu. (b) The landmark (map) of traditional medicine distribution and collection of different types of plants. (c) District map showing the collection site of plants from Kalrayan hills (Salem), (d) Pachamalai hills (Thiruchirappalli), and (e) Mundanthurai (Tirunelveli) rich biodiversity hot-spot of the Western Ghats. (f) Kolli hills (Namakkal), (g) Javadi hills (Vellore), part of the Eastern Ghats, which is a mountain range that runs mostly parallel to the east coast of South India.

**Figure 2:**
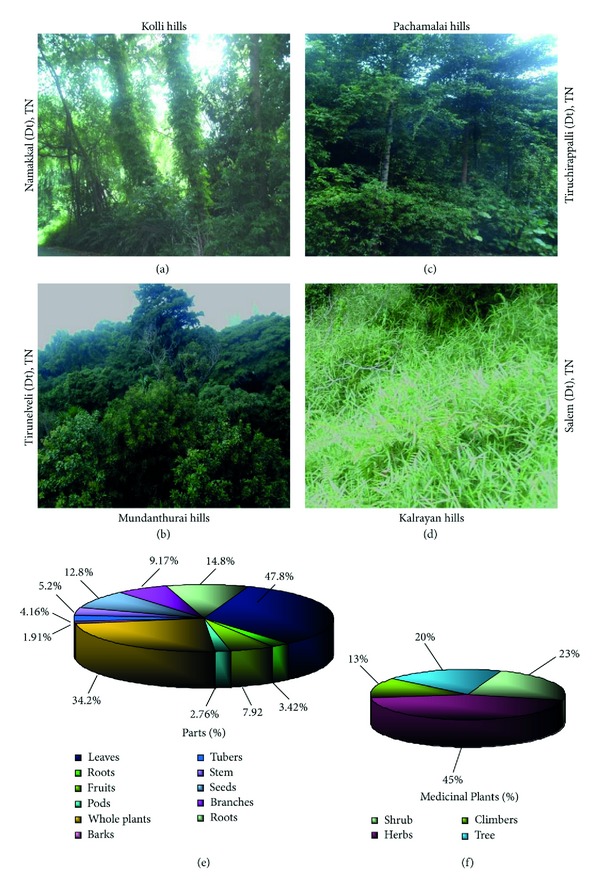
Diverse biodiversity richness of medicinal plants in Western and Eastern Ghats. (a) Topography of plant covering area in Kolli hills (Namakkal district, Tamil Nadu). (b) Aerial view of occurrence of medicinal plants in Mundanthurai hills (Tirunelveli district, TN). (c) Pachamalai hills (Trichy district) and its natural vegetation inhabitants for Malaiyali tribes. (d) Deforestation of natural herbal resources due to urbanization in Kalrayan hills (Salem district) in the Eastern Ghats of TN. (e) Medicinal plants and its various parts used by the natives (traditional healers) for the treatment of diverse human illness with a very high percentage of leaves and whole plants often used for herbal drug preparation by the local practitioners. (f) Various category of plants like shrub, herb, climbers and tree, and the parts used in medicine.

The Western and Eastern Ghats were selected for the present study with the cite map showing the landmarks (Figures 1(a)-1(b)). Kalrayan hills are situated north of Attur taluk (Salem district), one of the major range of hills in the Eastern Ghats of Tamil Nadu (Figure 1(c)). Pachamalai hills are situated to the north of Thuraiyur taluk of Tiruchirappalli district. The rich biodiversity part of Eastern Ghats lies between latitudes 11°09′ 00′′ to 11° 27′ 00′′ N and longitudes 78°28′ 00′′ to 78° 49′ 00′′ E, and occupies an area of about 527.61 square km. It is located near 11° 11′N 78°21E/11.18°N 78.35°E/11.18; 78.35 (Figure 1(d)). Mundanthurai is located nearly 45 km west of Tirunelveli district, TN, between latitude 8° 25′ and 8° 53′ N and longitude 77° 10′ and 77° 35′ E. This is the only area of Western Ghats that has the longest raining period of about 8 months and forms the catchment area for 14 rivers and streams (Figure 1(e)). Kolli Malai is a small mountain range located in Namakkal district. The mountains are about 1000–1300 m in height and cover an area of approximately 280 km. The Kolli hills are part of the Eastern Ghats, which is a mountain range that runs mostly parallel to the east coast of Tamil Nadu in South India (Figure 1(f)). Javadi hills are one of the largest in the Eastern Ghats in Vellore district in the northern part of the state of Tamil Nadu. They consist of bluish gray hills, with peaks averaging 3600–3800 feet or 1100–1150 meter (Figure 1(g)). Based on the vegetation type (Figures 2(a)–2(d)), the study area consists of (i) dry, deciduous, (ii) moist deciduous, and (iii) rain forests and diverse proportion of plant parts in abundance (Figures 2(e)-2(f)). Three different types of tribes (i.e., Kani, Malayali and Paliyan tribes) inhabit in the hill ranges. The Kani tribes, located at Mundanthurai, raise different types of vegetables in their own fields, while the Malayali tribes cultivate rice. They all engage not only in the agricultural work but also are involved in silvicultural work assigned by the forest department, Government of TN, India.

### 3.2. Medicinal Plants Glory

Western Ghats (Mundanthurai) and Eastern Ghats (Kolli hills, Javadi hills, Kalrayan hills, Pachamalai hills) possess a rich diversity of medicinal plants that are used as food and drug by different groups of tribal communities. Urbanization, habitat degradation, and fragmentation of these forests have resulted in the depletion of natural resources on which these tribes used to depend for their livelihoods. It has become increasingly difficult for them to live in their traditional way. In addition, the impact of modernization and urbanization has encroached in and around tribal settlements, thus changing their lifestyles. 

### 3.3. Plants Valued as Edibles

 Various types of plant parts are collected during different seasons, cooked, and eaten along with boiled rice (Table 1). For example, *Solanum nigrum* leaf is most commonly used in all the four regions. There are a large number of wild edible fruits, including yielding plants such as *Citrus acida, Ficus benghalensis, Ficus microcarpa*, *Ficus racemosa, Phyllanthus emblica*, *Solanum trilobatum,* and *Syzygium cumini *are popularly used by the tribes.

### 3.4. Plants Used for Snakebite Treatment

Thirty-four plants used for snakebite treatment are documented (Table 1). Snakebite is a major health hazard that leads to high mortality in tribal settlements. The majority of the antidotes are prepared freshly from plant materials frequently collected from the leaves of *A. paniculata, A. echioides, Aristolochia indica, E*. *alba*, *E. prostrata, M. pudica, O. sanctum, T. involucrata*, and *Cleistanthus collinus* (Oduvanthalai); the whole plants of *Achyranthes aspera *and* Wedelia calendulacea*; the stem-barks and nuts of *Strychnos nux-vomica*; the roots of* Hemidesmus indicus, Tephrosia purpurea, Rauwolfia serpentina, C. roseus, *and so forth, and the tubers of *Gloriosa superba*. The tuber paste is usually applied externally on the site of snakebite, and decoction is given orally for treatment by indigenous people. Besides, these tribes rely on the medicinal plants as ingredients for fabricating a kind of medicated stone for health management. “Vishakallu” (poison stone) is used by the indigenous groups called Kani in Kerala, India, to treat a snakebite. When the stone is placed directly on the bitten area, it sticks to the body to absorb the poison and then become detached when absorption seems to be complete. The ingredients of Vishakallu stones are made with leaves of *Ocimum sanctum, Anisomeles malabarica, Leucas aspera*, Piper betle, *Santalum album,* and the pebbles collected from the river bank. 

### 3.5. Survey of Medicinal Plants and Their Health Care Values

**Figure 3:**
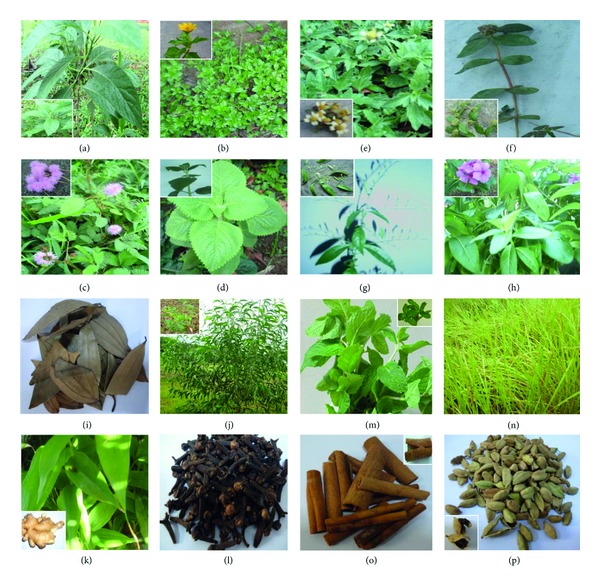
Medicinal aromatic herbs, spices, and toxic plants were collected from the tribal areas of the Western and Eastern Ghats region in Tamil Nadu, India. (a) *A. vasica* Nees (leaf), (b) *Eclipta alba* (L.) Hassk. (whole plant), (c) *Mimosa pudica* L. (whole plant), (d) *P. amboinicus* (L.)* Spreng.* (whole plant), (e) *T. procumbens* (L.) (whole plant), (f) *Euphorbia hirta* Linn (whole plant), (g) *A. paniculata * Wallich ex Nees, (H) *C. roseus* (L.) G.Don. (whole plant) used for therapy. (i) *Cinnamomum iners *Reinw. ex Blume (leaf) (j) *E. globulus* Labill. (leaf and bark), (k) *Z. officinale Rosc.* (Rhizome), (l) *E. caryophyllus (Sprengel) Bullock and Harrison *(flower buds), (m) *M. piperita * L. (whole plant), (n) *C. citratus *(DC.) *Clitoria ternatea* L. (whole plant), (o) *C. zeylanicum Garcin ex Blume* (bark), (p) *Elettaria cardamomum* White et Mason (fruit pod) used for medicine and food preparation.

The present study is an attempt to provide scientific basis and obtain justification for the traditional beliefs of reliance on a rich diversity of ethnomedicinal plants, along with the rich heritage of traditional medicine practices related to health care system made available by the primitive tribal communities located at different settlements. The native traditional practitioners called “vaidyars” have a good knowledge about the traditional plants locally available for treatment of various diseases (Figures 3(a)–3(p)). Such traditional medical knowledge is used for preparing home remedies, ill health prevention, and routine health maintenance. This knowledge is also applicable to cover other sectors of social life. During the ethnobotanical survey, the wealth of 78 medicinal plant species used by the indigenous tribal community for various types of health treatment was documented. The botanical names, family names, parts used, chemical constituents, and their application are provided (Table 1).

### 3.6. Antimicrobial Activity of Crude Extracts

In this study, we reported the antimicrobial screening of methanolic crude extracts of 78 medicinal plants (Table 2). Results revealed that 68 plant extracts displayed potent activities against one or more Gram-positive and -negative bacteria. Of which, *Tragia involucrata, Citrus acida, Aegle marmelos, Adhatoda vasica, Calotropis procera, Andrographis paniculata* and *Mentha piperita, Azadirachta indica, Sphaeranthus indicus,* and *Elettaria cardamomum* showed the highest antibacterial activity against the multidrug resistant *B. pseudomallei* (KHW and TES) and *S. aureus* at 100 *μ*g/mL concentration. The extracts showed pronounced antibacterial activity with their inhibitory zones ranging from 20 to 31 mm in diameter as compared to the standard drugs chloramphenicol and ceftazidime (29–33 mm). The majority of the plants demonstrated a powerful antimicrobial potency against the multidrug resistant strains of *B. pseudomallei* (KHW and TES), *K. pneumonia, *and *S. aureus.* Approximately, twenty-one plant extracts exerted only a weak or moderate effect against the tested bacteria, while the crude extract of 13 plants failed to show any effect at all. Except for the plant extracts of *T. involucrata, A. lanceolata, A. vasica*, and *S. indicus* extracts, the majority of the plant extracts were ineffective against the *V. damsela *infection, fascinatingly, only 11 plants exhibited activity against *P. aeruginosa, *of which* S. indicus, M. piperita, *and *C. procera *were found to have very strong inhibition of bacteria at the tested concentrations. Interestingly, sixteen plants such as *Andrographis echioides, C. auriculata, C. viscose, C. gigantea, T. arjuna, Oldenlandia umbellata, Boerhavia erecta, *and* E. hirta *exerted a strong activity against the Gram-positive *S. aureus* bacteria.

### 3.7. Phytochemical Screening of Plants

The results obtained from the phytochemical screening as shown in Table 1 indicate the presence of various types of secondary metabolites such as polyphenols, tannins, saponins, alkaloids, and glycosides/polysaccharides. Most of the plant extracts relatively rich in alkaloids, phenols, flavonoids, polyphenols, tannins, sterols, and terpenoids were found to inhibit the growth of organisms.

### 3.8. Antimicrobial Activity of Fractioned Compounds

**Figure 4:**
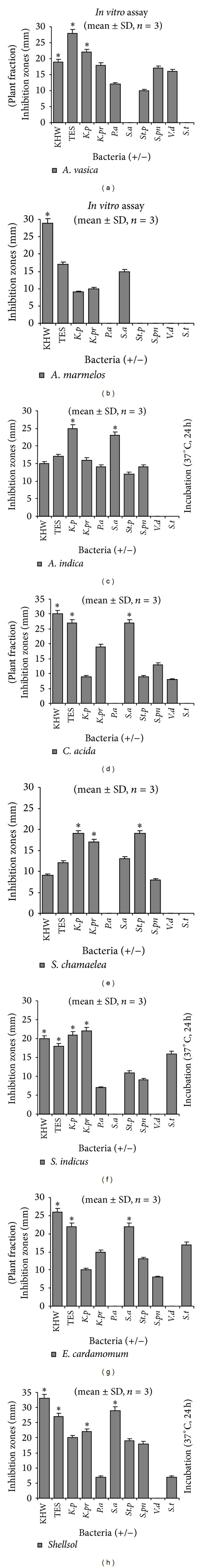
*In vitro* antimicrobial activity of purified fractions from the most active plant extracts tested against bacteria. Growth inhibition zones were measured and analyzed with mean ± standard deviation (SD), (*n* = 3) using one way analysis of variance. Level of significance at (*P > 0.01). Most of the fractions exerted a potent inhibitory effect against multidrug resistant Gram-negative bacteria (*B. pseudomallei* strains KHW and TES), *K. pneumonia,* and Gram-positive bacteria *S. aureus*.

**Figure 5:**
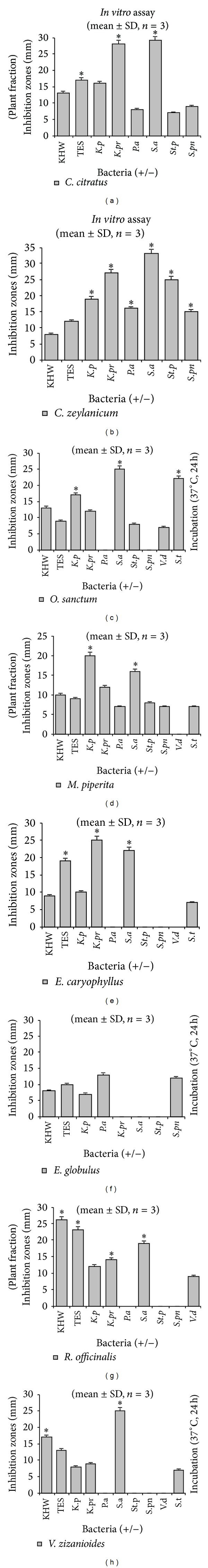
Comparison of antimicrobial effect of plant compounds obtained from the most popularly used sources of essential oils as assayed by the disc-diffusion method *in vitro*. It displayed a powerful activity against *B. pseudomallei* and *S. aureus* than the other bacteria strains. Other compounds showed only a moderate or weak action against the tested bacteria. Values for zone of bacterial growth inhibition were presented as mean ± SD, (*n* = 3) with level of significance at (*P > 0.01).

Active components were purified from the most active extracts for further testing. The compound shellsol of *T. involucrata *and *C. acida *exhibited the most potent action against the antibiotic resistant strains of *B. pseudomallei* (KHW),* S. aureus, B. pseudomallei* (TES), and *K. pneumoniae. A. marmelos* was also found to inhibit the growth of* B. pseudomallei* (KHW) more effectively than other tested bacteria.* A. vasica* showed the broad spectrum growth inhibitory activity on *B. pseudomallei* (KHW), *K. pneumoniae, K. pneumoniae,* resistant * B. pseudomallei * (TES), *S. pyogenes,* and *V. damsela. *However, *E. cardamomum *displayed antimicrobial activity on some of the *B. pseudomallei* (KHW), *S. pyogenes, B. pseudomallei *(TES), and *S. typhi* strains. Similarly, *A. indica* exerted the growth inhibition on *K. pneumoniae *and *S. aureus. *Remarkably, *Sebastiania chamaelea *was more active against* K. pneumoniae, K. pneumoniae, *and *S. pyogenes. *The compound from *S. indicus *inhibited the growth of* K. pneumoniae, K. pneumoniae, B. pseudomallei* (KHW and TES), and *S. typhi *strains, as compared to the activity shown by the crude extracts (Figure 4). The antimicrobial efficacy of fractions collected from the oil yielding plants was also compared with that of the tested compounds. *C. zeylanicum *and *R. officinalis *were the most sensitive in controlling the growth of *B. pseudomallei* (KHW), *S. aureus, K. pneumonia,* and* S. pneumoniae*. Fascinatingly, all the compounds obtained from aromatic plants, except those from *E. globules, *were found to be very effective against the multidrug resistant human pathogen *B. pseudomallei* (KHW) that causes melioidosis. On the other hand, compounds from *C citrates, O. sanctum, E. caryophyllus,* and *Z. zizanioide, *showed some promising effect only against *S. aureus* (Figure 5). The activity of the compounds were pronounced more than that of the oil yielding plant fractions.

### 3.9. Minimum Inhibitory Concentrations (MICs)

The antibiotic potential of the purified fractions was obtained from the MIC determination. The hydrocarbon ester shellsol (*T. involucrata*) and *C. acida* showed an interesting inhibitory potential against *S. aureus *(MIC of 7.8 *μ*g/mL) and* B. pseudomallei* strain of KHW (MIC of 15.6 *μ*g/mL). *A. vasica* showed an MIC of 15.6 *μ*g/mL against *B. pseudomallei* (KHW) and an MIC of 31.25 *μ*g/mL against *K. pneumoniae, K. pneumoniae, S. pyogenes, *and *V. damsela *strains. Fractions from *A. marmelos* and terpenoid from *A. indica *exerted bacteriostatic effect with MIC values of 31.25 *μ*g/mL on some selected bacteria including *B. pseudomallei* of KHW,* S. aureus, *and *B. pseudomallei* of TES. The MIC of 31.25 *μ*g/mL was found for* E. cardamomum *against *S. aureus*, *K. pneumonia, *and *S. pyogenes*. *S. indicus *displayed a very strong inhibition against MDR* K. pneumoniae *(MIC of 15.6 *μ*g/mL), and against *B. pseudomallei* (KHW and TES) at MIC of 31.25 *μ*g/mL. When the antimicrobial efficacies of purified fractions from aromatic plants were compared, the *C. zeylanicum* fraction displayed an important antimicrobial effect against *S. aureus *(MIC of 7.8 *μ*g/mL), MDR *B. pseudomallei* of KHW (MIC of 15.6 *μ*g/mL), and *S. pneumoniae *(MIC of 31.25 *μ*g/mL). The essential oil from *M. piperatea* showed MIC value of 31.25 *μ*g/mL against *K. pneumoniae, S. aureus,* and *B. pseudomallei* (KHW), respectively. *O. sanctum *and *C. citratus *fractions also showed antimicrobial activity (MICs of 31.25–125 *μ*g/mL) only at higher concentrations against the tested bacteria. In addition to that, higher concentrations (>250 *μ*g/mL) (of *Vetiveria* fractions) were required to inhibit *Vibro species,* and others (including *E. globulus* fractions) failed to show any effect at tested concentrations (7.8–125 *μ*g/mL). However, the purified fractions (from most active medicinal plants) showed strong bacteriostatic inhibition against the tested organisms (Table 3).

### 3.10. Cytotoxic Effects of Plants

 When the components were assayed for cytotoxicity against the normal human skin fibroblasts (HEPK) cells, the compounds obtained from *E*. *cardamomum*, *T*. *involucrata*, *S*. *indicus*, *C*. *acida*, *A*. *vasica*, *A*. *marmelos*, *A*. *indica,* and *A*. *paniculata* did not show toxicity up to 1000 *μ*g/mL (see Figures S1 and S2 in Supplementary Material available online at http://dx.doi.org/10.1155/2013/525613). A slight reduction of cell proliferation was noted only at higher doses (2000 *μ*g/mL). In contrast, cell proliferation was markedly reduced after exposure of HEPK cells to *O*. *sanctum*, *E*. *globulus*, *V*. *zizanioides*, *C*. *citratus,* and *E*. *globulus* compounds. There was no gradual reduction in skin cell proliferation seen after exposure to *C*. *zeylanicum*, *R*. *officinalis,* and *M*. *piperita* (see Figures S3 and S4) compounds. The toxicity was found to be concentration-dependent when the skin fibroblasts (HEPK) cells were exposed to various compounds. The cell proliferation was increased by the influence of the plant components at the lower concentrations. Whereas the oil yielding plant compounds showed inhibition of cell proliferation and toxicity at higher doses 250–1000 *μ*g/mL.

## 4. Discussion 

The Western Ghats is considered as one of the richest biodiversity hotspots in the world [41]. In this survey, we collected nearly 78 medicinal plants from Western and Eastern Ghats that are edible and popularly used for curing various ailments including snakebite. Traditional remedies have a long-standing history in many tribal settlements in TN, India, and they continue to provide useful and applicable tools for treating ailments [42]. The ingredients that make up the “Vishakallu” stone, which is used as an antidote for snakebite, are different herbs and pebbles available from the river banks. Likewise, aqueous paste and decoction obtained from the leaves of *A. paniculata* are widely used for snakebite treatment by indigenous people [43]. Previous studies have reported that ethnomedicine plays major roles in conserving the disappearing knowledge of tribal communities [44–47]. The traditional beliefs of reliance on a rich diversity of ethnomedicinal plants located at different settlements have also been confirmed in another study [48, 49]. Herein, we explored the various types of traditional practices reported by the primitive tribal communities with a view to gain further knowledge from such studies. 

In the present investigation, potentially rich sources of tribal medicine (71 plants) were scientifically evaluated for their antibacterial activity against the MDR bacteria, and the accumulated data was disseminated for the first time to the scientific community. Out of the 71 medicinal plants screened for the antibacterial activity, 10 of them (*T. involucrata, C. acida, A. marmelos, A. vasica, C. procera, A. paniculata* and *M. piperita, A. indica, S. indicus, *and *E. cardamomum*) displayed the highest antibacterial activity against the multidrug resistant *B. pseudomallei* (KHW and TES) and *S. aureus* strains. The antibacterial activity of those crude plant extracts was as equally effective as that of the standard drugs. Our findings corroborated with the previous reports made on the *antistaphylococcal* activity of tribal medicinal plants [34, 50, 51]. On the other hand, isolated components from the most active extracts of *T. involucrata*, shellsol, and *C. acida *exhibited the most potent action against the antibiotic resistant *B. pseudomallei* (KHW),* K. pneumoniae*, and *S. aureus *strains. These results further confirmed our previous findings on the leaves of *T. involucrata* and its compounds hydrocarbon ester-like shellsol, which displayed a high antibacterial effect against the different bacterial strains, especially that of *S. aureus* [34]. Eugenol and caryophyllene are the active agents contained in the *M*. *piperita* [52] and *O. sanctum *plants, which are believed to be mainly responsible for the antimicrobial properties of these plants [53]. Interestingly, the compounds obtained from the aromatic plants such as *C. zeylanicum *and *R. officinalis* were also found to be very effective against the multidrug resistant human pathogen *K. pneumoniae, S. aureus*,* S. typhi, *and *B. pseudomallei* (KHW) that causes melioidosis.

The inhibitory potential determined for the shellsol (*T. involucrata*), vaseline (*A. vasica*), *C. acida, *and* C. zeylanicum* indicates that the MIC of 7.8–31.25 *μ*g/mL found against the* B. pseudomallei* of KHW, *K. pneumoniae, K. pneumoniae, S. pyogenes, *and* V. damsel, *and S. *pneumoniae *was quite low. Similarly, lower MIC values were found for *M*. *piperita* (MIC of 1.13–2.25 mg/mL) against the above bacterial strains [52]. MIC was found for the most active alcohol extracts of *A. salvifolium *(MIC 0.034–0.263 mg/mL) on *S. aureus* [54], and the MIC for *S. trilobatum *aqueous extracts determined against the tested organisms ranged from 0.06 to 0.5 mg/mL [55]. *C. zeylanicum* was found to have an effective antibacterial activity (MIC 64 *μ*g/mL) against *P. aeruginosa, E. coli, B. subtilis, *and* S. aureus *[56]. Previously, several investigators have demonstrated that active agents exert interesting activity against bacteria even at lower concentrations tested [57, 58]. The bacteriostatic mechanism involves damage to the cell walls of bacteria, followed by inhibition of protein synthesis that ultimately leads to bacterial death [59]. The most active plants are widely used by various tribes as traditional treatment (i.e., cut wounds, skin infection, and scabies), thus indicating the potential for further development into promising drugs. In order to ascertain the safety and efficacy of the most active compounds, their effect on human skin fibroblast cells was evaluated. Chemical constituents of *E. cardamomum, T. involucrata, S. indicus, C. acida, A. vasica, A. marmelos, A. indica, *and *A. paniculata* plants failed to produce any noticeable toxicity up to 1000 *μ*g/mL. Although some of the tested compounds exhibited a slight reduction of cell proliferation and some minor morphological changes, such changes were insignificant at lower doses and became evident only at higher doses. However, certain aromatic compounds of *O. sanctum, E. globulus, V. zizanioides, C. citratus, *and* E. globulus* plants showed reduction of cell proliferation against HEPK cells.

 Our phytochemical screening also provides evidence of the presence of several types of compounds that are mainly responsible for the remarkable antibacterial effect of these plants. The differences noted for the bactericidal activity of various plant extracts in this study appears to be directly related to the diversity of compounds (shown in parentheses) that are accumulated in the following plants (e.g., *A. marmelos* and *O. umbellate*) [60–62]. Compounds like tannins, phenol, and polyphenols can bind the Gram-negative bacteria to form a heavy soluble complex on the cell surface, which subsequently disturbs the availability of receptor on cells and kills the bacteria [63]. Several species having wide spectra of antimicrobial activity mainly due to the active constituents such as essential oil, phenolic compounds like thymol, carvacrol in oregano and thyme, eugenol in clove, and cinnamon were also identified previously [64, 65]. Essential oils degrade the cell wall, interact with the cell components, and then disrupt the cytoplasmic membrane [66]. The antimicrobial effect of phenolic compounds may involve multiple modes of action, including damage to the membrane protein, interference with membrane integrated enzymes [67], causing leakage of cellular components, coagulation of cytoplasm, depletion of the proton motive force, alteration of fatty acid and phospholipid constituents, impairment of enzymatic mechanisms for production and metabolism, alteration of nutrient uptake and electron transport [68], influencing the synthesis of DNA and RNA, and destroying protein translation and the function of the mitochondrion in eukaryotes [69]. The mode of action of antimicrobial agents depends on the type of microorganism and is mainly related to their cell wall structure and the outer membrane arrangement. Most of the plant spices and herbs contain complex phenolics (i.e., phenolic acids, flavonoids, tannins, lignans, coumarins, quinines). In addition, the mechanisms of action of each phenolic compound against various bacteria are also very complicated [26, 70]. Further investigation is therefore required to understand the relationship between the antimicrobial action and the chemical structure of every phenolic compound in the tested extracts. The information available from previous pharmacological sources combined with the findings herein reported on the medicinal plant extracts may serve as essential data for future drug development to combat diseases caused by the MDR bacterial strains. 

## Supplementary Material

Evaluating herbal drugs in vitro could be a valuable tool for screening antibiotic potential of plants. To develop new strategy for improvement for the assessment of their pharmacological, toxicological profile, scientific evidence based approaches are being employed to appropriately evaluate composition, quality, potential medicinal activity and safety of these natural products.

## Figures and Tables

**Table 1:** Some of the important traditional medicinal plant species, families, voucher specimens, parts used, yield of extracts, phytochemical screening, and toxicity on human macrophage cells.

Scientific name	Family	Voucher specimen	Plant parts	Yield (gm)	Phytochemical analysis
*Adhatoda vasica* Nees	Acanthaceae	D2020	Leaf	6.4	Vasicine
*Aegle marmelos* (L.) Correa ex Roxb.	Rutaceae	D2018	Root-bark	5.8	Alkaloids
*Alangium salvifolium* (L.) f. Wangerin.	Alangiaceae	0140	Leaf	6.3	Phenolic
*Andrographis paniculata * Wallich ex Nees	Acanthaceae	0061	Leaf	6.8	Andrographolide
*Andrographis echioides * Nees	Acanthaceae	0116	Leaf	7.0	Terpenoids,
*Acalypha indica* L.	Euphorbiaceae	29644	Leaf	6.1	Acalyphe
*Acalypha lanceolata* L.	Euphorbiaceae	15791	Leaf	7.1	Alkaloids
*Achyranthes aspera* L.	Amaranthaceae	2666	Leaf	2.7	Betaine
*Ageratum conyzoides* L.	Asteraceae	4812	Leaf	3.7	Essential oils
*Asteracantha longifolia* L.	Acanthaceae	0234	Stem	7.4	Glycosides
*Azadirachta indica* A. Juss.	Meliaceae	D0204	Leaf, bark	6.7	Tannins
*Borassus flabellifer* L.	Arecaceae	D0202	Root	0.8	Flavonoids, phenolics
*Boerhavia erecta* L.	Nyctaginaceae	10897	Whole plant	2.8	Phenolics
*Calotropis procera* (Ait.) Ait. f.	Asclepiadaceae	D073	Root-bark	1.3	Terpenoids
*Calotropis gigantea* (L.) R.Br.ex Ait	Asclepiadaceae	D070	Milky latex	4.8	Alkaloids
*Cassia auriculata* L.	Caesalpiniaceae	0141	Leaf	4.9	Saponins
*Cassia occidentalis* L.	Caesalpiniaceae	0111	Root	9.5	Flavonoids, saponins
*Cassia tora * L.	Caesalpiniaceae	0100	Stem, bark	6.9	Saponins
*Cassia fistula * L.	Caesalpiniaceae	037	Whole plant	7.9	Saponins
*Cardiospermum halicacabum* L.	Sapindaceae	0125	Whole plant	5.8	Flavonoids
*Catharanthus roseus* (L.) G.Don.	Apocynaceae	0029	Leaf, root	1.4 0.7	Alkaloids
*Cinnamomum zeylanicum Garcin ex Blume *	Lauraceae	00209	Bark	3.1	Essential oil, Tannin
*Cinnamomum iners* Reinw. ex Blume	Lauraceae	043-c	Leaf, bark		Alkaloids
*Cissus quadrangularis* Roxb.	Vitaceae	D02023	Leaf	6.8	Glycosides
*Citrus acida* Roxb. Hook.f.	Rutaceae	0213	Leaf	5.2	Saponins, Terpenoids
*Centella asiatica* (L.)	Umbelliferae	0138	Whole plant	8.9	Flavonoids, Alkaloids
*Clerodendrum inerme* (L.) Gaertn.	Verbenaceae	D02043	Stem	7.8	Sterols, diterpenes
*Clitoria ternatea* L.	Papilionaceae	D02026	Seed	9.8	Protein
*Cleistanthus collinus * (Roxb.) Benth. and Hook.f.	Euphorbiaceae	0011	Whole plant	0.03	Cleistanthin, collinusin
*Cleome gynandropsis* L.	Capparidaceae	12247	Leaf	6.2	Glycosides
*Cleome viscose* L.	Capparidaceae	29999	Leaf	2.7	Phenolics
*Coccinia grandis* W & A	Cucurbitaceae	D02030	Leaf, root	0.9	Glycosides
*Cymbopogon citratus* (DC.)	Gramineae	D012	Root	0.25	Essential oil
*Datura metel* L.	Solanaceae	D02038	Leaf, stem	3.9	Steroids
*Eucalyptus globulus *Labill.	Myrtaceae	D0220	Leaf	1.2	Terpenoids
*Eclipta alba* (L.) Hassk	Asteraceae	D028	Whole plant	0.7	Phenolic
*Euphorbia hirta* Linn	Euphorbiaceae	0018-c	Whole plant	0.12	—
*Eclipta prostrata * (L.)	Asteraceae	D210	Leaf	1.10	Triterpenoid, saponin
*Eugenia caryophyllus *(Sprengel) Bullock & Harrison	Myrtaceae	0025	Flower buds	1.16	Essential oils
*Elettaria cardamomum White et Mason *	Zingiberaceae	0009	Fruit pods	3.17	Essential oils
*Gloriosa superba* L.	Liliaceae	020-S	Tuber	1.08	Alkaloids, phenol
*Jatropha curcas* L.	Euphorbiaceae	015	Whole plant	5.3	Alkaloids, flavonoids
*Hyptis suaveolens* (L.) Poit.	Lamiaceae	24688	Leaf	6.3	Essential oil
*Hemidesmus indicus* L.	Asclepiadaceae	D-009	Roots		Coumarins
*Ichnocarpus frutescens* (L.) R.Br.	Apocynaceae	0110	Root, flower	7.3	Terpenoids
*Leucas aspera* (Willd.) Link	Labiatae	0114	Leaf	8.3	Triterpenes
*Lawsonia inermis* L.	Lythraceae	T261	Leaf	0.9	Glycosides, phenolic
*Madhuca longifolia* (L.) JF Macbr	Sapotaceae	D01415	Nut	9.3	Sitosterol
*Merremia hastate L. *(Desr.) Hallier.f.	Convolvulaceae	10894	Whole plant	4.0	Alkaloids
*Mentha piperita* L.	Lamiaceae	0217-c	Whole plant	0.7	Essential oils
*Morinda tinctoria* Roxb	Rubiaceae	0122	Leaf	1.4	Glycosides
*Mimosa pudica* L.	Mimosaceae	0071	Whole plant	0.6	—
*Oldenlandia umbellata* L.	Rubiaceae	D02047	Leaf	4.4	Alkaloids
*Ocimum sanctum* L.	Lamiaceae	0016	Whole plant	3.0	Alkaloids
*Piper attenuatum* Buch. Hamex Miq.	Piperaceae	007	Flower	4.6	Alkaloids
*Plumbago zeylanica* (L.) Cav	Plumbaginaceae	0121	Root	4.8	*Plumbagin *
*Plectranthus amboinicus* (L.) *Spreng. *	Lamiaceae	0410	Whole plant	1.2	Essential oils, terpenoids
*Phyllanthus debilis *L. (Klein ex Willd)	Euphorbiaceae	0120	Whole plant	4.9	Polyphenol
*Phyllanthus madraspatensis* L.	Euphorbiaceae	0117	Whole plant	5.0	Polyphenol
*Premna tomentosa* Willd.	Verbenaceae	0129	Leaf	5.3	Diterpenes
*Rosmarinus officinalis * L.	Lamiaceae	0017	Root	0.23	Essential oils
*Rauwolfia serpentine* L.	Apocynaceae	020-S	Root	1.15	Alkaloid
*Sebastiania chamaelea* (L.) Muell Arg.	Euphorbiaceae	0034	Leaf	1.1	Polyphenol
*Solanum trilobatum* L.	Solanaceae	D02054	Leaf, flower	4.0	Tannins
*Sphaeranthus indicus* L.	Asteraceae	D02060	Whole plant	1.0	Essential oil
*Swertia chirata* (L.) Ham.	Gentianaceae	D0540	Whole plant	1.6	Glycodises
*Strychnos nux-vomica* L.	Loganiaceae	S-22	Nuts	0.36	Alkaloids
*Tragia involucrata * L.	Euphorbiaceae	D068	Leaves	1.6	Shellsol
*Tinospora cordifolia* (Willd.) Miers ex Hoof.f & Thoms	Menispermaceae	0118	Leaf, root, stem	5.0	Glycosides, tannins
*Tridax procumbens* L.	Compositae	10649	Leaf	1.8	Flavonoids
*Terminalia arjuna* (DC) W & A	Combretaceae	033-c	Bark	8.0	Phenolics
*Tephrosia purpurea* (L.) Pers	Fabaceae	S-43	Whole plant	0.8	Isoflavone
*Vitex negundo* L.	Verbenaceae	0031	Leaf	2.4	Terpineol
*Vetiveria zizanioides* L.	Gramineae	0051	Root	1.03	Essential oil
*Withania somnifera* (L.) Dunal	Solanaceae	D02063	Root	2.1	Alkaloids
*Wedelia calendulacea* Less	Asteraceae	S-24	Leaves		Flavonoids
*Zingiber officinale* Rosc.	Zingiberaceae	0327	Rhizome	2.3	Tannins
*Zanthoxylum limonella* (Dennst.) Alston	Rutaceae	009	Bark	1.9	Alkaloids, essential oil

Class of chemical compounds: A: alkaloids, S: saponins, T: tannins, St: steroids, G: glycosides, T: terpenoids, P: polyphenol, P: phenolics, Sh: shellsol, H: hydrocarbon esters.

**Table 2:** Antimicrobial activity of methanol extract of aromatic medicinal plants and essential oils evaluated against multidrug resistant (MDR) human pathogens at 100 *µ*g/mL concentration.

Scientific name	Microorganisms; growth inhibition zones (6 millimeter in diameters)									
	*KHW*	*TES*	*K.p*	*K.pr*	*P.a*	*S.a*	*St.p*	*S.p*	*V.d*	*V.d*
*Adhatoda vasica* Nees	28	19	22	18	12	—	10	17	16	—
*Aegle marmelos* (L.) Correa ex Roxb.	29	17	9	10	—	15	—	—	—	—
*Alangium salvifolium* (L.) f. Wangerin.	15	10	8	8	—	12	9	10	—	—
*Andrographis echioides* L.	12	9	—	—	—	17	8	9	—	—
*Andrographis paniculata* Wallich ex Nees	26	21	19	13	—	25	8	16	12	—
*Acalypha indica* L.	—	—	—	—	—	18	—	—	—	—
*Acalypha lanceolata* L.	—	—	12	13	—	10	—	—	21	—
*Achyranthes aspera* L.	—	—	7	14	—	15	12	11	—	—
*Ageratum conyzoides* L.	10	8	—	—	—	—	—	—	—	—
*Asteracantha longifolia* L.	16	12	—	—	—	15	—	—	—	—
*Azadirachta indica* A. Juss.	15	17	21	16	14	23	12	14	—	—
*Borassus flabellifer* L.	9	10	17	8	—	—	9	10	—	—
*Boerhavia erecta* L.	—	—	8	9	7	16	—	—	—	—
*Calotropis procera* (L.)	—	—	15	—	18	28	9	—	—	—
*Calotropis gigantea* (L.) R.Br.ex Ait	11	9	—	—	—	20	—	—	9	8
*Cardiospermum halicacabum* L.	23	—	—	14	—	9	19	—	—	—
*Catharanthus roseus* (L.) G.Don.	13	7	12	9	—	15	11	8	—	—
*Cassia auriculata* L.	17	13	12	—	—	19	13	—	—	—
*Cassia occidentalis* L.	18	—	—	—	—	—	—	—	—	—
*Cassia tora* L.	—	—	—	—	—	—	—	—	—	—
*Cassia fistula* L.	—	—	—	—	—	—	—	—	—	—
*Citrus acida* Roxb. Hook.f.	26	22	—	12		29	9	13	8	—
*Cissus quadrangularis* L.	—	—	—	—	—	—	—	—	—	—
*Cinnamomum zeylanicum Garcin ex Blume *	14	16	7	20	—	22	19	7	—	—
*Cinnamomum iners * Reinw. ex Blume	20	16	—	15	—	16	12	—	—	—
*Rosmarinus officinalis * L.	—	9	—	10	—	7	—	8	—	—
*Centella asiatica* (L.)	9	8	—	—	—	11	—	—	—	—
*Clerodendrum inerme* (L.) Gaertn.	13	7	12	—	—	15	11	8	—	—
*Clitoria ternatea* L.	—	—	—	—	—	—	—	—	—	—
*Clitoria ternatea* L.	16	12	—	13	—	8	10	—	—	—
*Cleome gynandropsis* L.	—	—	12	19	11	—	9	15	12	—
*Cleome viscose* L.	—	—	8	10	14	20	—	—	—	—
*Coccinia grandis* W & A	9	10	17	8	—	—	9	10	—	—
*Cymbopogon citratus* (DC.)	16	18	—	17	—	14	—	—	—	—
*Datura metel* L.	—	—	—	—	—	—	—	—	—	—
*Eclipta alba* (L.) Hassk	20	—	—	9	—	—	10	—	—	—
*Euphorbia hirta* Linn	11	—	—	—	—	16	—	—	—	—
*Eucalyptus globulus* Labill.	—	—	—	7	—	—	—	7	—	—
*Eugenia caryophyllus* Bullock & Harrison	—	7	—	11	—	9	—	8	—	—
*Elettaria cardamomum* White et Mason	21	20	7	14	—	22	12	17	—	—
*Hyptis suaveolens* (L.) Poit.	8	10	8	7	—	—	—	—	—	—
*Ichnocarpus frutescens* (L.) W.J. Aiton	—	—	—	—	—	—	—	—	—	—
*Jatropha curcas* L.	—	—	—	8	—	11	—	12	—	7
*Leucas aspera* (Willd.) Link	9	10	17	8	—	12	9	10	—	—
*Lawsonia inermis* L.	—	—	—	—	—	—	—	—	—	—
*Madhuca longifolia* (L.) JF Macbr	18	16	14	19	12	—	8	7	9	—
*Merremia hastate* L. (Desr.) Hallier.f.	—	—	—	—	—	—	—	—	—	—
*Morinda tinctoria* Roxb	—	—	—	—	—	—	—	—	—	—
*Mentha piperita* L.	23	17	26	12	20	25	19	—	—	—
*Ocimum sanctum * L.	12	9	11	7	—	15	7	8	—	—
*Oldenlandia umbellata * L.	—	—	—	—	—	17	—	—	—	—
*Piper attenuatum* Buch. Hamex Miq.	13	7	12	21	—	17	11	8	—	—
*Plumbago zeylanica* (L.) Cav	9	10	17	8	—	12	9	10	—	—
*Plectranthus amboinicus* (L.) Spreng.	—	—	—	8	11	15	—	—	—	—
*Phyllanthus debilis * L. (Klein ex Willd)	7	8	—	9	—	9	18	7	—	—
*Phyllanthus maderaspatensis* L.	17	—	—	—	—	—	—	—	—	—
*Premna tomentosa* Willd.	13	10	—	15	—	9	10	—	—	—
*Gloriosa superba* L.	17	16	7	8	—	15	—	—	—	8
*Sebastiania chamaelea* (L.) Muell Arg.	19	12	—	17	—	13	19	8	—	—
*Solanum trilobatum* L.	—	—	13	9	—	8	—	—	—	—
*Sphaeranthus indicus* L.	20	18	21	7	22	—	11	9	—	16
*Swertia chirata* (L.) Ham.	—	—	—	—	—	—	—	—	—	—
*Terminalia arjuna* (W. & A)	—	—	—	—	—	16	—	—	—	—
*Tinospora cordifolia* (Willd.) Miers ex Hoof.f & Thoms	—	—	23	16	—	12	15	—	—	—
*Tridax procumbens* L.	9	8	7	—	—	14	—	—	—	—
*Tragia involucrata* L.	25	23	20	—	—	31	28	22	19	—
*Vitex negundo* L.	—	—	—	—	—	14	—	—	—	—
*Vetiveria zizanioides* (L.)	11	9	7	7	—	16	—	—		8
*Withania somnifera* (L.) Dunal	12	20	—	—	—	15	12	—	—	—
*Zingiber officinale* Rosc.	14	11	7	15	—	7	12	7	—	—
*Zanthoxylum limonella* (Dennst.) Alston	13	7	12	9	—	—	11	8	—	—
Chloramphenicol (30 *µ*g/disc)	21	12	15	17	29	16	15	18	13	11
Ceftazidime (30 *µ*g/disc)	33	16	22	19	16	25	21	20	12	15

*Bacteria (+/−). Results obtained in the disc diffusion assay; antibacterial activity is expressed as the mean ± SD (*n* = 3), of the inhibition by the extract and its diameter around the discs. One way analysis of variance was performed (mean ± SD, *n* = 3 replicates). Size of inhibition zones were including the sterile blank discs 6 millimeter (mm) in diameters. Absence of bacterial inhibition indicates (—), antibiotic disc (30 *µ*g/disc).

**Table 3:** Minimum inhibitory concentrations (MICs) of purified plant fractions and essential oils against antibiotic resistant bacteria.

Botanical name	Family	Parts used	Gram-positive and -negative bacteria (MICs *µ*g/mL)								
			*KHW*	*TES*	*K.p*	*K.Pr*	*S.a*	*St.p*	*S.p*	*V.d*	*S.t*
*A. indica Juss. *	Meliaceae	Seed (fraction)	31.25	62.5	125	<250	31.25	—	—	—	—
*A. marmelos* (L.)	Rutaceae	Root-bark (F)	31.25	31.25	—	—	31.25	62.5	125	250	—
*A. paniculata* Nees	Acanthaceae	Leaf (fraction)	250	—	125	—	62.5	—	—	125	—
*A. vasica* Nees	Acanthaceae	Fraction (Stem)	15.6	31.25	31.25	62.5	—	<250	31.25	31.25	—
*C. acida* Roxb.	Rutaceae	Leaf (fraction)	15.6	62.5	—	—	7.8	—	31.25	—	—
*E. cardamomum* White et Mason	Acanthaceae	Fraction (WP)	62.5	—	31.25	—	31.25	250	62.5	—	—
*S. indicus* (L.)	Euphorbiaceae	Whole plant (F)	31.25	125	15.6	—	62.5	—	—	<250	—
*T. involucrata* (L.)	Euphorbiaceae	Shellosol (leaf)	15.6	31.25	—	—	7.8	62.5	—	—	—
*Cinnamomum zeylanicum* (L.)	Lauraceae	Bark (fraction)	15.6	62.5	—	—	7.8	31.25	—	—	125
*Cymbopogon citratus* (L.)	Graminae	Leaf (fraction)	250	<250	62.5	—	62.5	125	—	—	—
*Eugenia caryophyllus* (L.)	Myrtaceae	Flower buds (F)	62.5	125	—	—	62.5	—	—	—	—
*Eucalyptus globulus* (L.)	Myrtaceae	Fraction (leaf)	—	—	—	—	—	—	<250	—	<250
*Mentha piperita* (L.)	Labiatae	Fraction (WP)	—	62.5	31.25	—	31.25	—	<250	—	—
*Ocimum sanctum* (L.)	Labiatae	Leaf (fraction)	62.5	125	—	31.25	62.5	—	125	<250	—
*Rosmarinus officinalis* (L.)	Labiatae	Rosemary oil	31.25	—	—	—	31.25	—	—	—	—
*Vetiveria zizanioides* (L.)	Graminae	Root (fraction)		—	125	125	—	—	—	>250	>250

The bacterial growth inhibitory activity was compared for significant differences within the bacterial strains by broth-dilution method at 250, 125, 62.5, 31.25, 15.6, and 7.8 *µ*g/mL. F: fractions.

## Abbreviations

MDR: Multidrug resistant bacteria
TES: Strain of *Burkholderia pseudomallei*
MRSA: Methicillin-resistant *Staphylococcus aureus*
VRE: Vancomycin-resistant enterococci
MTB: *Mycobacterium tuberculosis*
CH_3_OH: Methanol
CFU: Colony forming unit
HCl: Hydrochloric acid
TLC: Thin layer chromatography
H_2_SO_4_: Sulphuric acids
UV: Ultraviolet spectrophotometer
HEPK: Human skin fibroblasts.
