# Supplementary figures and images for: Evaluation of Aromatic Plants and Compounds Used to Fight Multidrug Resistant Infections
Source: Evid Based Complement Alternat Med. 2013 Oct 8;2013:525613. doi: 10.1155/2013/525613 (PMC3816052; doi:10.1155/2013/525613)

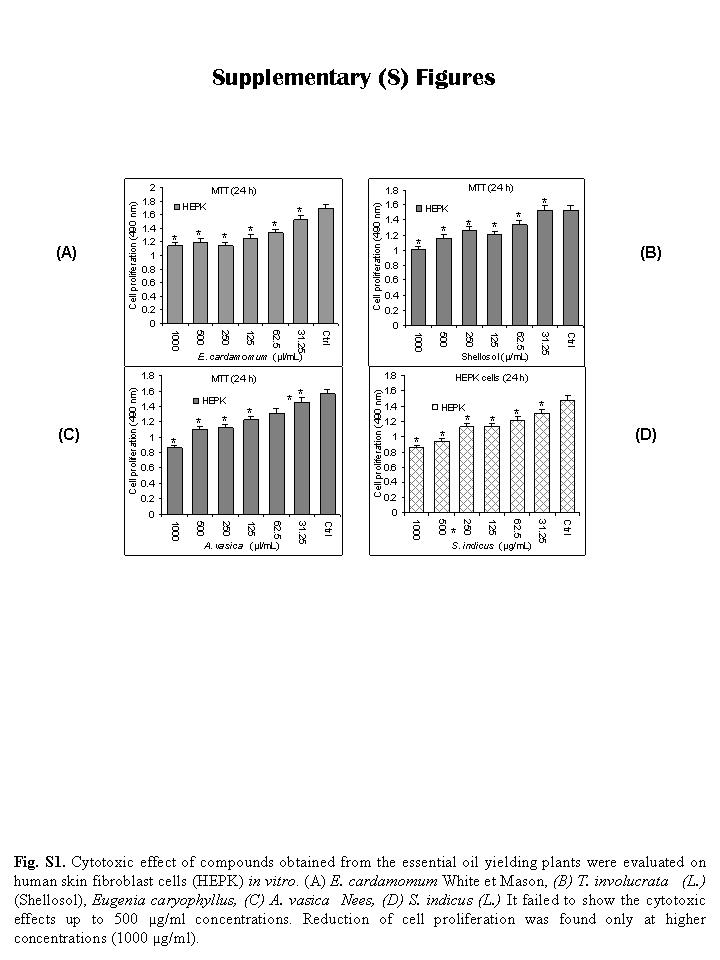

Supplement: Supplementary file 1 — Evaluating herbal drugs in vitro could be a valuable tool for screening antibiotic potential of plants. To develop new strategy for improvement for the assessment of their pharmacological, toxicological profile, scientific evidence based approaches are being employed to appropriately evaluate composition, quality, potential medicinal activity and safety of these natural products. [file 525613.f1.zip › Fig S1.jpg]

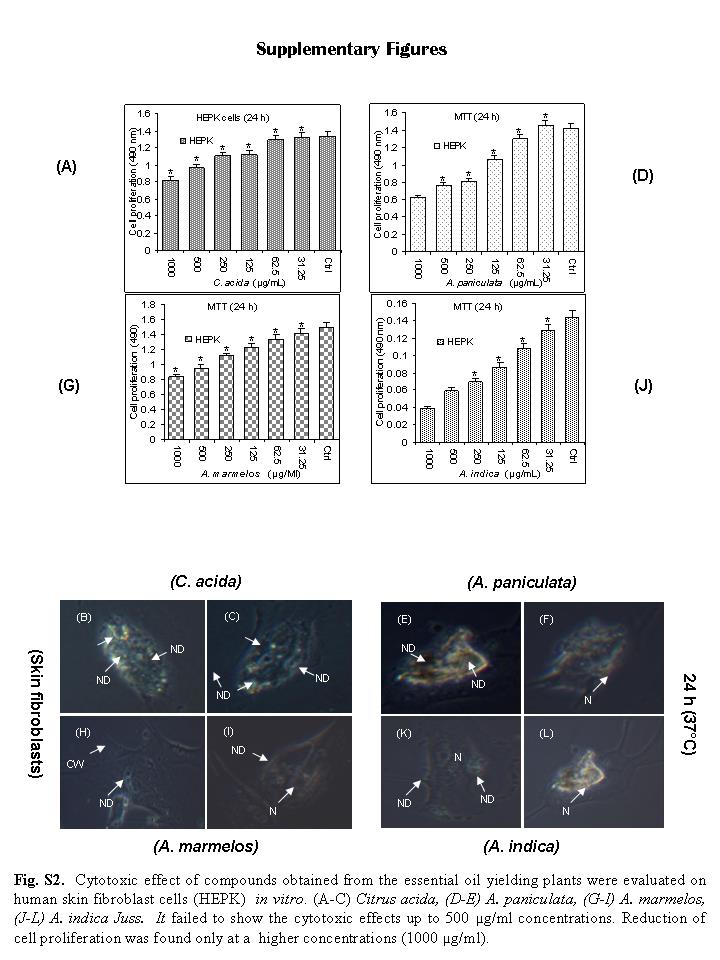

Supplement: Supplementary file 1 — Evaluating herbal drugs in vitro could be a valuable tool for screening antibiotic potential of plants. To develop new strategy for improvement for the assessment of their pharmacological, toxicological profile, scientific evidence based approaches are being employed to appropriately evaluate composition, quality, potential medicinal activity and safety of these natural products. [file 525613.f1.zip › Fig S2.jpg]

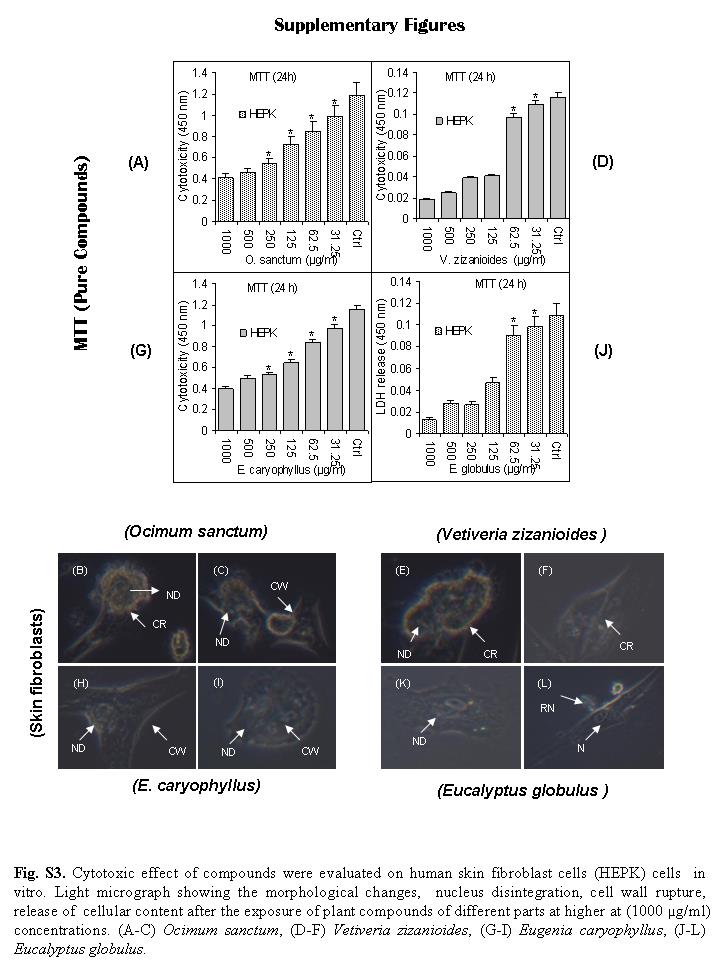

Supplement: Supplementary file 1 — Evaluating herbal drugs in vitro could be a valuable tool for screening antibiotic potential of plants. To develop new strategy for improvement for the assessment of their pharmacological, toxicological profile, scientific evidence based approaches are being employed to appropriately evaluate composition, quality, potential medicinal activity and safety of these natural products. [file 525613.f1.zip › Fig S3.jpg]

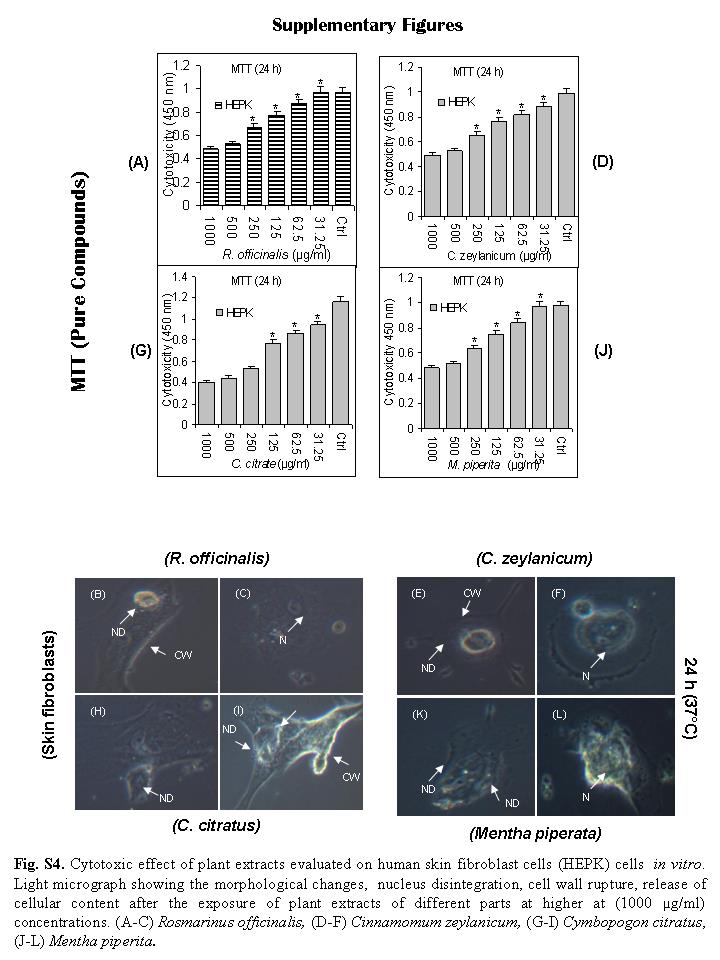

Supplement: Supplementary file 1 — Evaluating herbal drugs in vitro could be a valuable tool for screening antibiotic potential of plants. To develop new strategy for improvement for the assessment of their pharmacological, toxicological profile, scientific evidence based approaches are being employed to appropriately evaluate composition, quality, potential medicinal activity and safety of these natural products. [file 525613.f1.zip › Fig S4.jpg]
